# Dogs in the Wroclaw Stronghold, 2nd Half of the 10th–1st Half of the 13th Century (Lower Silesia, Poland)—An Zooarchaeological Overview

**DOI:** 10.3390/ani11020543

**Published:** 2021-02-19

**Authors:** Aleksandra Pankiewicz, Krzysztof Jaworski, Aleksander Chrószcz, Dominik Poradowski

**Affiliations:** 1 Institute of Archaeology, University of Wroclaw Szewska 48, 50-139 Wrocław, Poland; aleksandra.pankiewicz@uwr.edu.pl (A.P.); krzysztof.jaworski@uwr.edu.pl (K.J.); 2 Department of Biostructure and Animal Physiology, Faculty of Veterinary Medicine, Wroclaw University of Environmental and Life Sciences, Kożuchowska 1, 51-631 Wrocław, Poland; aleksander.chroszcz@upwr.edu.pl

**Keywords:** dog, early Middle Ages, archaeozoology, animal use, social role of animals

## Abstract

**Simple Summary:**

Over the centuries of coexistence between humans and dogs, both the appearance of our four-legged companions and their social perception have changed. This article aims at identifying the most probable morphological and functional types of dogs found in Poland in the period from the 10th to the first half of the 13th century. The authors will also try to address the issue of how dogs were treated in the early Middle Ages and what social and economic roles these animals played. These considerations are based on the remains of several dozen dogs discovered in the medieval Wroclaw stronghold, one of the most important centres in Poland at the time. We will use finds from other archaeological sites in Poland and written sources concerning this part of Europe. It has been proven that specific “breeds” of dogs were found in this area. Their appearance and size were probably related to the specific function of the quadrupeds. Dogs were treated very differently in the early Middle Ages: Both as a companion for the elite, and as a source of skins, bones, and even meat.

**Abstract:**

This article pertains to the issue of early medieval dogs (10th–mid-13th century) from the territory of Poland and Central Europe. The study is based on dog remains from the Wroclaw Cathedral Island (Ostrów Tumski), one of the most important administrative centres of early medieval Poland, the capital of a secular principality and the seat of diocese authorities. The main morphological and functional types of dogs living in Wroclaw and other parts of Poland were characterized on that basis. It has been concluded that the roles and perceptions of dogs were very ambiguous. On the one hand, they were hunting companionship for the elite and were considered a symbol of devotion and loyalty. On the other hand, dogs symbolised disgrace. In everyday life, these animals were sometimes abused, their skin was sometimes tanned and their bones modified into tools, and in exceptional cases, dogs were even eaten.

## 1. Introduction

The dog was the earliest animal domesticated by humans, accompanying us through thousands of years of shared history. Although the dog was the first domesticated species, it is difficult to establish the exact historical period when this process took place. The only way to do that, if it is possible at all, seems to be through archaeological, archaeozoological, biogeographic, and genetic studies [1]. Archaeozoological analyses of animal bone remains face significant difficulties in differentiating the early forms of the domestic dog from its wild ancestor. Recent studies have shifted this date even earlier, as there is evidence of late Pleistocene pet dog remains in modern Belgium, the Czech Republic, and southern Siberia [2,3,4]. Apart from the chronology of domestication, the appearance of dogs, their function in society (guard, shepherd, and hunting dogs), and the perception of the four-legged companions changed as a result of the process.

Dog domestication resulted in morphological, physiological, and behavioural changes in the animal through deliberate breeding and human-controlled natural selection [5]. Contemporary dog breeds are the result of many years of human activity based on deliberate interference in the natural selection of animals, consolidating characteristics desired by the breeder and eliminating those considered redundant or unfavourable [6]. Although most of the breeds known to us originated in the modern era, there are also some that have a much longer lineage. There are examples of dogs with a specific morphological and functional type known already in antiquity [7,8,9,10]. While in many respects the Middle Ages were not a period that carried on the ancient achievements (including deliberate breeding), it seems obvious that dogs still performed various functions and thus differed in terms of appearance and temperament [11].

This article aims at determining which types of dogs were most common in Poland in the early medieval period (2nd half of the 10th century–1st half of the 13th century). The authors will try to verify how different they were compared to today’s dogs.

In the second part, we will analyse the use of the dog’s remains as raw material. Although the use of dog hides or bones is often reported by early medieval researchers, it is not a popular topic of studies. Nowadays dogs are most often treated as a pet, guardian, shepherd dog, or a hunting companion, particularly in the European culture. These functions were also performed by early medieval dogs, but their social role might have been different, which we tried to verify in our study. Therefore, the analysis of the dogs’ bone remains was also used in an attempt to reconstruct the role of the domestic dog and the way these animals were perceived by medieval people.

So far, several studies have been devoted to the reconstruction of dogs that lived on the territory of Poland in the Middle Ages [12,13]. Most of the well-studied archaeological sites from that period have animal remains analyses, including dog bones [14,15,16,17,18,19,20,21,22,23,24]. Other studies (not only limited locally) focus on the cultural role of individual animals (including dogs) in the early Middle Ages [25,26,27,28]. However, only few publications try to comprehensively tackle all the above-mentioned issues. The aim of this article is to create such a cross-sectional study of early medieval dogs, covering both physical characteristics and the cultural role of these animals in Poland and Central Europe.

## 2. Materials and Methods

Animal bone remains identified as coming from domestic dogs from the early medieval stronghold on the Wroclaw Cathedral Island were used for the study. In particular, remains from the following sites were included:

1. Wrocław Cathedral Island, Katedralna 4 Street;
2. Wrocław Cathedral Island, św. Idziego (St. Giles) Street;
3. Wrocław Cathedral Island, trench VI.

The remains’ species affiliation was established based on a visual analysis using the osteological comparative material for modern dogs from the reference collection of the Division of Animal Anatomy, Faculty of Veterinary Medicine, Wroclaw University of Environmental and Life Sciences.

Whenever possible, an osteometric examination was performed on the dog remains to determine the morphological type of skull and the height at the withers [29,30,31,32,33,34].

Furthermore, the bone material was analysed for the presence of processing traces and its possible usage as tools. Examples of using other animal elements (i.e., dog hides), preserved fragmentarily in archaeological materials, were presented [35].

Both published and unpublished data from trenches I-II and III, located within the same site on Wrocław Cathedral Island, were also used. However, their usefulness for the purpose of this study was varied, as it included both detailed results on research focused on dogs [12] and those providing mainly statistical information [36].

Based on the available data, an attempt was also made to indicate the dogs role in the lives of medieval inhabitants of the Wrocław stronghold. The obtained results were compared with the available literature.

## 3. Results and Discussion

### 3.1. Bone Remains of Dogs from the Cathedral Island in Wrocław

Archaeozoological studies of animals’ skeletal remains from the Cathedral Island have been discussed numerous times [12,14,15,28,36]. The remains’ state of preservation directly influenced the number of identified specimens (NISP) and limited the possibility of inference on the basis of animal bones (i.e., the skull type definition, height at withers estimation, or pathologies and other visible marks identification).

**Figure 1:**
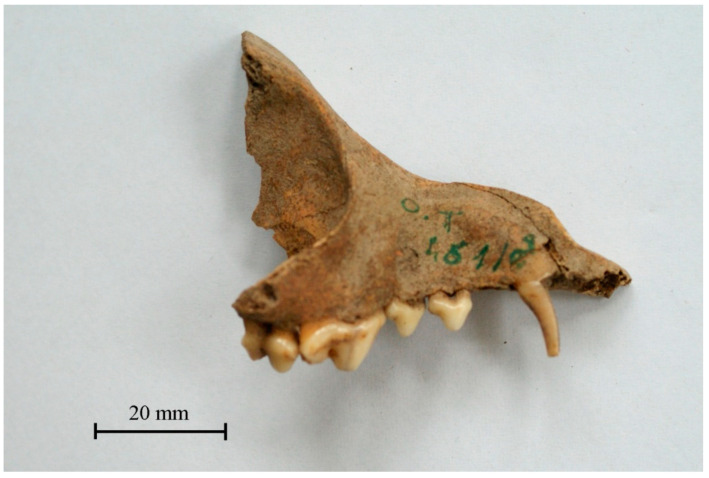
The dog’s skull fragment, Wrocław Cathedral Island, Katedralna 4 Street, according to [14]. Reproduced with permission from A. Chrószcz, Wratislavia Antiqua; published by Instytut Archeologii UWr, 2012.

**Figure 2:**
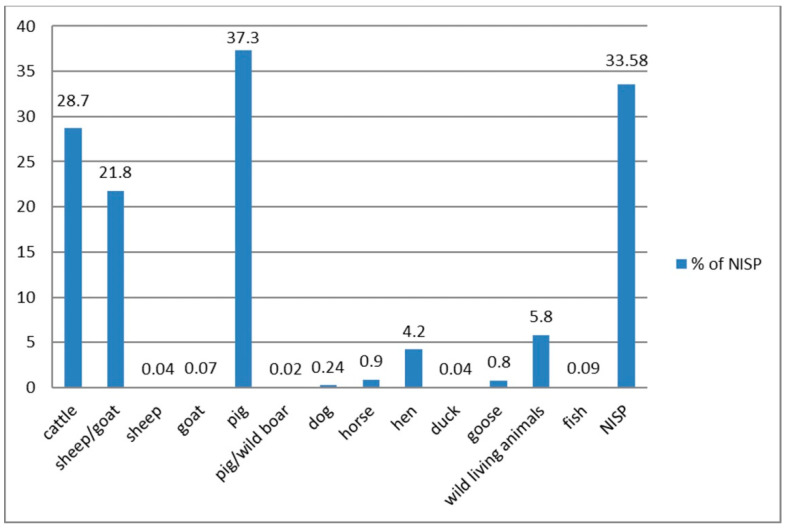
The frequency of the consumptive animal remains estimated on Wrocław Cathedral Island, św. Idziego (St. Giles) Street. NISP—number of identified specimens (NISP = 5568).

In the material from the Wroclaw stronghold, 460 bones were identified as part of a domestic dog [12,14,15]. Some of them have already been thoroughly analysed [23], others are known from an unpublished report [36] and recent publications [14,15]. It is estimated that the minimal number of individuals (MNI) was 70. However, during the settlement’s functioning from the late 10th to the first half of the 13th century, many more dogs probably lived in the area. Few skeletal remains of dog in the archaeozoological material are largely (Figure 1) due to the fact that they were not kept as animals for food (such as cattle, sheep, goats, or pigs), so their bones are much less often found in assemblages containing consumption waste (Figure 2).

**Figure 3:**
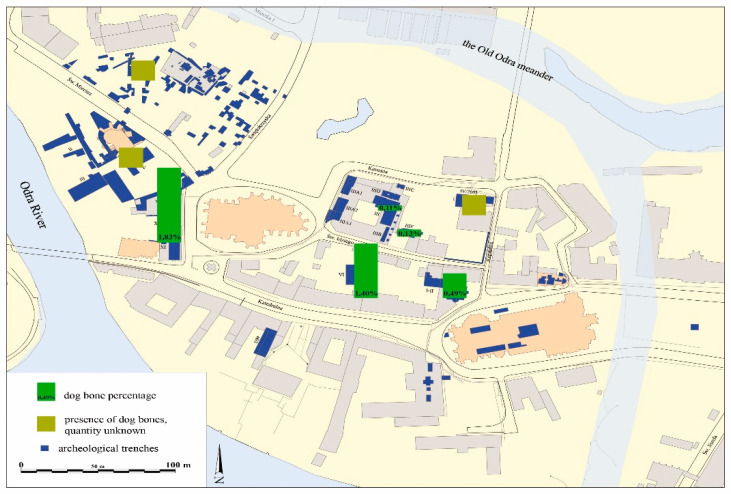
The stronghold in Wroclaw on Cathedral Island. Percentage of dog remains.

The share of dog bones on the Cathedral Island ranged from 0.1% to 1.8% of NISP (Figure 3). The disproportions usually result from the fact that in such a small collection, even several more significantly affect the percentage share. Moreover, the relation between the minimal number of examples and the minimal number of individuals (MNE/MNI) would greatly influence the result. This condition might not always be met in the archival studies, which could also result in discrepancies. However, they do not differ significantly from the percentage of dog skeletal remains in the frequency of species distribution in sites discovered in Wroclaw [24] and Polish territory [17,18].

**Figure 4:**
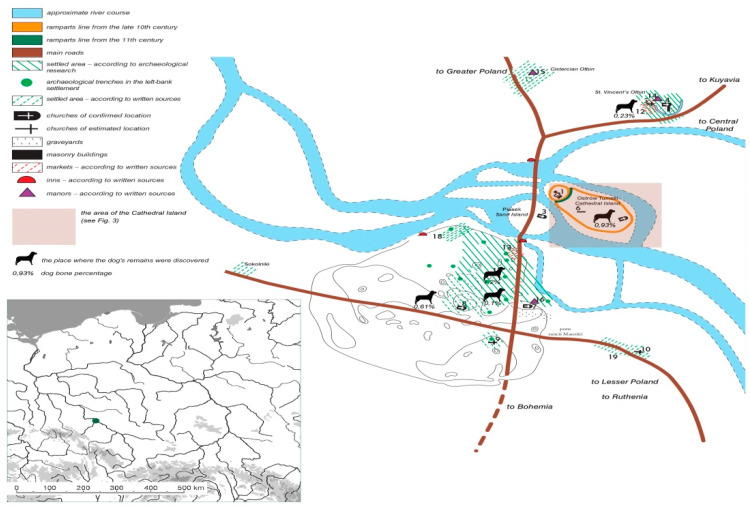
Wroclaw on the map of Poland and the early medieval settlement complex in Wroclaw around 1200 AD, according to [37]. Reproduced with permission from J. Pieklaski, C. Buśko, and J. Połamarczuk, the Historical Atlas of Polish Towns: Wroclaw; published by Instytut Archeologii i Etnologii PAN, 2017 (with modifications by A. Pankiewicz).

Moreover, dog remains identified in other early medieval sites in Wroclaw (Figure 4) [16,24] were not the subject of detailed metric analyses allowing us to define their morphological type and potential “breed”. In contrast to the remains from the Cathedral Island, they also often lack a precise chronological and cultural context, which lowers their cognitive value. These sites and other material collections containing dog skeletal remains from the area of present-day Poland will constitute a comparative base for Wroclaw material. To define the cultural importance of the dog, the authors will use examples from Central Europe, and, exceptionally, also from outside the Slavic region (Western Europe, Eastern Europe, Scandinavia).

### 3.2. Early Medieval “Breeds” and Their Status in the Light of Studies on Wroclaw Materials

One cannot talk about breeds as understood today in relation to early medieval dogs, as they differed from those known today [12,31]. However, it is possible to estimate height at the withers to some extent.

Thanks to the detailed research on dog skeletal remains from the early medieval Cathedral Island stronghold, we know what the local dogs looked like in the respect of skull morphology [12]. The remains of three size groups were discerned. The first consisted of small, short-snapped, spitz-like dogs with a much longer head and converging frontal crests. Their height at the withers was estimated at 35–45 cm. It was quite a popular “breed” in the early Middle Ages in Poland. The dogs whose remains were discovered in the nearby Opole (100 km to the east of Wroclaw), early medieval Kruszwica (Kuyavia region) [17], and within the Tum settlement near Łęczyca (central Poland) [18] were also described as ‘spitz-like’. Small dogs constituted 45% of the species population in the Cathedral Island stronghold. This group also includes a specimen with characteristically short limbs, which could resemble a modern dachshund [12], not a frequent discovery for the early medieval Poland. While examining dog skeletal remains from the same period found in Wroclaw and Opole, Wyrost [12] maintained that the Wroclaw specimen, dated to the 12th or 12th/13th century, is one of the oldest examples of such type in Poland. However, similar finds dated to the earlier La Tene period are known from the Kuyavia region [13].

The second largest group (31%) consisted of bones of medium-sized, brachycephalic animals that can be compared to modern pointers. They had about 55 cm at the withers [12]. Similar remains were discovered in Opole [12] and Kruszwica [17]. This group also includes specimens similar to Polish hounds. Bones of such animals were also discovered in Ostrów Lednicki (Greater Poland region) [18]. The least numerous (24%) group on Wroclaw is represented by relatively large, mesocephalic dogs similar to German Shepherds, with a height of about 65 cm at the withers [12]. Bones with a similar morphotype were also excavated in Kruszwica [17] and Ostrów Lednicki [18].

No remains of dogs resembling greyhounds [12], considered to be one of the oldest “breeds” already popular on the British Isles, were found on the Cathedral Island [26,38]. According to Wyrost [12], they appeared in Poland in later periods. However, similar remains are known from Tum near Łęczyca [19]. Their presence in this part of Europe is also evidenced by the skeletal discovery at the Chotěbuz-Podobora site (9th–10th century), located in the Czech Republic, but very close (approx. 350 m) to the Polish border (north-western outskirts of the border town of Cieszyn) [39].

In the case of worse preserved bone remains, when it was not possible to establish a comparable modern breed, we defined the specimen height at the withers. Earlier findings regarding the height of dogs were confirmed by the recent skeletal material studies from the Wroclaw Cathedral Island [15]. They do not differ significantly from the results obtained in other sites. Medium dogs were the most prevalent in Opole [12], Kruszwica (up to 55 cm at the withers) [17], Ostrów Lednicki (51 cm to 61.7 cm) [18], and Poznan (43.6 cm to 60.8 cm, average 52 cm) [21]. Small dogs were present, but not numerous in Kruszwica (10%), similarly to large (about 65 cm tall) and very large specimen, with a height at the withers above 70 cm (20% each) [17] According to Makowiecki [13], dog height on the territory of Poland ranged from 22.6 cm to 76.7 cm. The average values at the withers are similar: 54.1 cm with some regional deviations, e.g., dogs from the Greater Poland region were slightly taller than in Silesia [13].

Based on dog remains from Wroclaw archaeological sites dating back to the late Middle Ages (2nd half of the 13th–15th/16th century), individuals of the species were generally smaller, with a height at the withers ranging from 29.66 cm to 49 cm [16,40,41]. Similar observations were made in relation to late medieval and early modern dogs from other parts of Poland. This may be related to a fashion for smaller ‘breeds’, but also result from practical reasons. Smaller dogs were probably preferred in cities (where most of the analysed dog bones come from), as they were easier to keep in tight buildings [13].

It is also worth noting that a significant number of dog remains from the Wroclaw stronghold belong to young (2–3 years old), or slightly older (4–7) specimens [12], which means that the animals did not live long. However, the analyses carried out for the Cathedral Island did not indicate any lesions that could indicate maltreatment [12,14,15]. However, it was not always the case. Archaeozoological studies from Western Europe has shown that dog remains extremely often show signs of fractures, which may be related to mistreatment or abuse by humans [42,43].

Skeletal material from other animals provides evidence that dogs often consumed food leftovers thrown away by humans. It could show a way of managing organic waste or concern for domesticated canines. Traces of gnawing are often found in osteological material excavated in larger Polish sites. They are most often associated with the activities of dogs [15,16,18,19,22,23] and probably their deliberate feeding by the owners.

### 3.3. Dog Remains as Raw Material

**Figure 5:**
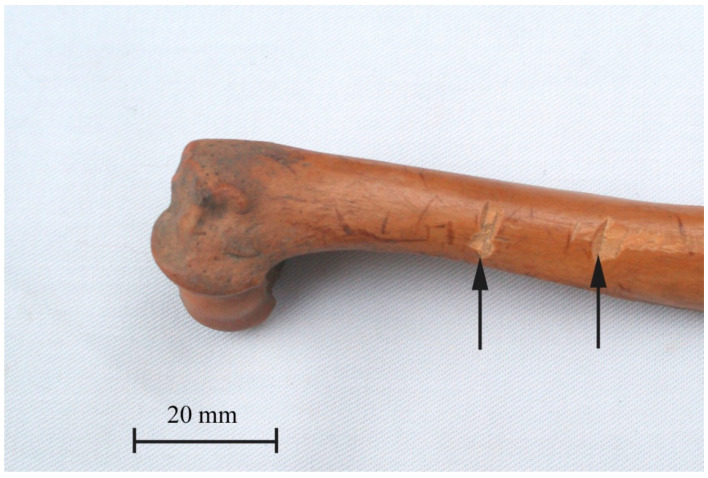
The humerus of dog with visible cut-marks, Wrocław-Nowy Targ site according to [16]. Reproduced with permission from A. Chrószcz, Wratislavia Antiqua; published by Instytut Archeologii UWr, 2018.

Based on studies of canine remains from older periods (from the Neolithic to the Roman times), it was established that dogs were occasionally consumed at times [31,44,45,46,47,48,49,50]. In The Middle Ages, humans also reached for this source of protein under certain circumstances [51]. However, researchers agree that it rarely happened [12,15,16,18,29]. It is believed that cynophagy occurred only in times of food shortage, such as hunger or siege [52]. The analysis of bone remains from Wroclaw sites confirms these observations, as no traces of meat portioning were found [12,15]. Individual cuts on dog bones (Figure 5) were discovered in Wrocław-Nowy Targ [16], as well as in other settlements in the Polish territory [19], but their interpretation is ambiguous and does not indicate consumption.

Tanning dog hides was not popular, but it happened. On the Wroclaw Cathedral Island, the share of predatory animal hides was 5.3%, but it was not indicated that they were obtained from dogs [35]. However, products made of this raw material were found in other sites, e.g., vest elements [53], gloves, or other small items of clothing [29]. There are also examples of spinning and weaving dog hair [29].

**Figure 6:**
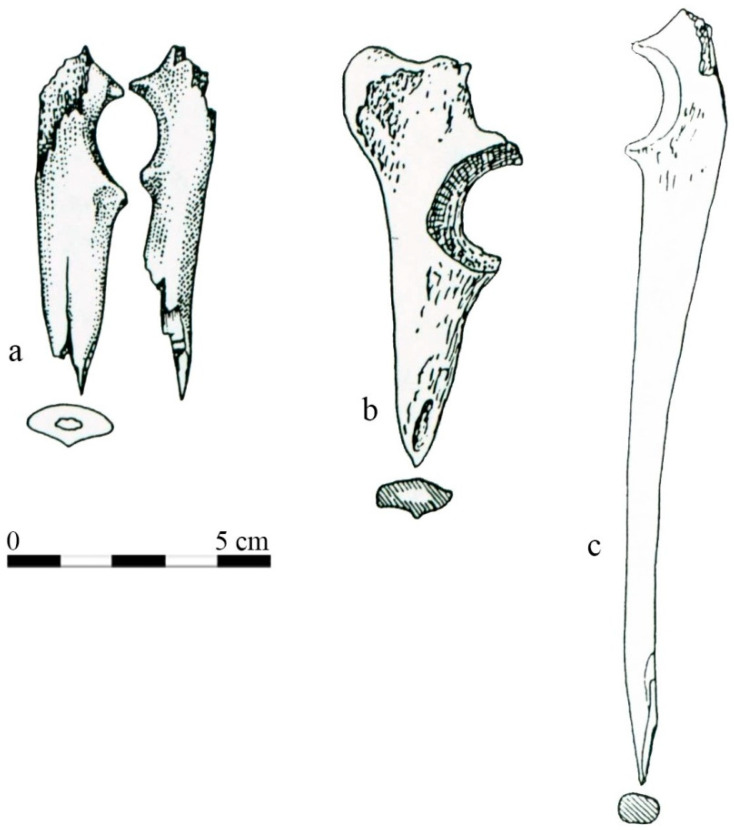
Points made of dog ulna from the Cathedral Island in Wroclaw, (**a**–**c**) According to [54]. Reproduced with permission from K. Jaworski, Wytwórczość i użytkowanie wyrobów z kości i poroża na wrocławskim Ostrowie Tumskim w X-XV wieku (Ph.D. thesis); University of Wroclaw, 1993.

Dog bones were also used to produce everyday items, but they were the least commonly processed raw material among all the domestic animals. So far, only 8 such artefacts have been discovered on the Cathedral Island. All of them were discovered in the central part of the stronghold, north of the cathedral [54]. More recent excavations have not produced such findings [55,56,57]. In fact, only points (called in archaeology as bone spikes) and antler artefacts used for piercing, engraving, knitting, etc. (Figure 6).

Points made of dog bones from the Wroclaw stronghold were discovered in older layers, dating from the 4th quarter of the 10th century to the mid-11th century. Items made of dog bones clearly come from (more or less) one chronological horizon [54]. Such artefacts were not found in the younger layers, which could be potentially explained by the lower population of dogs in the stronghold in the second half of the 11th century and later, but the low quantity of dogs’ bone material prevent the stating of any hypothesis. On the contrary, most of the unprocessed dog bones were found in the younger layers [12,36,54]. These observations could show that, over time, dogs’ raw material was withdrawn from use on the Cathedral Island. Probably in later period, the sheep and goat metapodia were more frequently used for the production of such items, like points, etc. Moreover, the ethical and moral reasons for giving up dog-based raw material cannot be ruled out [54].

Dog skeletal artefacts were made of 4 types of bone. Ulna was most common—3 items, then radial and femoral bones—2 items each. One item was made from a metatarsal or metacarpal bone. The same unprocessed bones were found during excavations both in the eastern and western part of the Cathedral Island [12,14,15]. However, they were not numerous, and of the several hundred dog remains discovered so far, most of them were skulls, mandibles, and teeth, while limb bones were much less common [12,14,54]. Similar trends can be observed in other sites [19].

The usage of dog limb bones for the production of points is also known from other settlements in Poland. Early medieval layers in Łęczyca and Kołobrzeg contained spikes made of dog tibia [19,22]. At the latter site, only one dog bone artefact had a function other than spikes—a bone-like frame made of an undefined bone [22].

Besides bones, the soft tissue of dogs could also have a specific purpose. For many centuries, folk medicine considered dog fat as an excellent remedy for lung ailments: Tuberculosis and asthma. It has also been used to treat jaundice. Dog’s blood was used to ward off demons, and faeces mixed with water were used as a remedy for stomach ulcers [58]. However, these uses cannot be confirmed based on archaeological materials.

### 3.4. The Function of Dogs in the Early Middle Ages

In relation to all eras, one of the basic functions of dogs is guarding private belongings, as well as herding and protecting other animals [12,13,29]. In medieval centres such as the Cathedral Island, the latter function was not used, as it is assumed that animal husbandry was not performed for commercial purposes (or at least it was not the main source of income) among city inhabitants. There was no room to keep numerous animal herds within the densely developed island, and its inhabitants served the ruler, or later the Church [59]. Guard dogs were a different matter, which is probably why they were kept at homes despite the scarce space. Medium and smaller dogs identified in the excavated bone material [12] were also suitable for that purpose.

The literature also points to using dogs in sleds. No skeletal changes were observed in the remains from Wroclaw that could indicate such a function. In other sites, carrying and pulling loads was commonly reflected in degenerative lesions in the spine and joints [42]. Information on such use of dogs is also provided by early medieval written sources, such as Al-Marwazi’s account of the transport of various goods from Kamska Bulgaria using dog sleds [29].

The use of dogs for hunting is also described in historical sources. In the discussed period, the activity was regulated by the princely regale and performed by the elites [60]. Hunting with dogs is widely mentioned in the chronicles from Western and Eastern Slavic lands, usually in the context of rulers and their entourage [61,62]. Dogs’ presence and role also influenced the names of settlements located near major centres of power (such as Psary near Wroclaw) [37,60]. It may indicate the existence of specialized dog breeding places operating under the obligation of the princely law [12,29,60].

The presence of individual “breeds” of dogs is also important. In the early Middle Ages, greyhounds, dachshunds, and pointers were considered to be an indicator of their owner’s social prestige [13,19]. Their appearance differed significantly from the more popular and common types similar to modern spitzes or German Shepherds. Dogs could also be used for hunting, and the remains of specimens comparable both to modern pointers and dachshunds (commonly used for that purpose) were found in the Cathedral Island.

Another, somewhat natural function of dogs was to provide companionship. Such a role may be reflected in their bone remains found in all major sites: Both in strongholds and settlements. It is also indicated by the deliberate selection of some “breeds”—more on that below.

### 3.5. Social and Symbolic Role of Dogs

Archaeological and archaeozoological evidence confirm the varied roles of the dog throughout history (companion, guardian, ritual, and sacrificial, as well as combat animals) [8,63,64,65,66,67,68,69].

The specific remains distribution may testify to the social and symbolic role of dogs. The shortage of dog bones in relation to the real number of animals living in a given settlement has already been emphasised. One can also note the overrepresentation of dogs’ skulls in relation to the rest of the skeleton. This may be related to ritual deposits in the form of animal skulls—usually a horse cranium was used for that purpose, but boar and dog skeletal remains were also deposited [29]. Dogs’ teeth could also be used as charms [70], which may be another reason for collecting their skulls.

Dog bone clusters on the Wroclaw Cathedral Island indicate that the animals may have been buried in the stronghold [15,37]. However, these findings are ambiguous and only suggest the accumulation of bones in one place [15]. However, examples of laying whole dogs in the ground are known from other sites. Several such finds were reported within the settlement in Łęczyca, as well as in Gniezno and Janów Pomorski. The animal skeletons were incomplete, so it is impossible to speak with full certainty about a careful burial within the stronghold. These finds were referred to as ‘deposits’ [19,20,23]. Both in the territory of Poland, the area of today’s Germany and Scandinavia, dogs also occur in sepulchral contexts. This applies to dog burials in the strict sense within settlements [42] and in cemeteries as a unique grave gift, most likely a sacrifice. This is evidenced by depositing in graves only a half or headless animal body, as well as a fairly high percentage of graves with dog remains in some cemeteries in these areas [27]. Such practices are also confirmed in written sources, especially in relation to the Rus and Scandinavian peoples [27,71]. The presence of animal necropolises already in antiquity has been confirmed in the literature, also including dog skeletal remains [8].

Written sources also shed light on the perception of dogs in the discussed period. These animals very often appear in the records of chroniclers, usually as hunting companions [26,60,61,62,71,72]. Since ancient times, a faithful dog that does not leave its master even after death has been a popular theme since antiquity, similarly to a dog pointing to the murderer of its owner. Similar topics also appear in early medieval sources, e.g., Isidore of Seville (7th century), Thietmar of Merseburg (11th century), Gerald of Wales (12th century), or Bartholomaeus Anglicus (13th century) [26,71,72]. These authors usually describe dogs as faithful and devoted animals. Such motifs may also be found in medieval iconography [15,73]. However, since it is mainly Western European, it will not be considered more broadly. Dogs were also attributed certain healing properties. Wounds licked by them were to heal significantly faster, which in the Middle Ages was also associated with the symbolism of renewing the soul through confession [72]. To this day, dog saliva is believed to have healing properties [58], although its composition helps mainly the animals themselves, not necessarily humans.

Based on the above information, it would seem that dogs’ image in the early medieval society of Central Europe was very positive. They were considered faithful companions both in life and death, and due to special breeding and participation in hunting, also associated with the elites. However, written sources from the period in many cases show that dogs also symbolised disgrace. In the Polish and Czech chronicles, puppies were put to the breasts of women instead of babies as a punishment for marital infidelity, but also a cruel way of treating the enemy [62,74]. Political opponents were humiliated by tying a scabby dog to their backs and exposing them to the public [74]. Sources also clearly indicate that calling someone a dog or comparing them to the animal was an insult [71,74], probably even more severe than today. Other describe dogs’ greed when devouring meat (including human carcasses) as well as eating their own vomit [62,72,73]. The latter act was supposed to symbolize returning to already committed sins [72].

Scandinavian mythology also includes stories putting dogs in bad light. Along with their demonic counterparts, they are intermediaries between worlds of the dead and the living [27], resembling Cerberus known from the Greek mythology. For medieval people, the difference between dangerous hyenas or wolves and friendly dogs is often blurred. Negative attitudes towards the latter could result from the fear of wolves, but also herds of feral dogs, also mentioned in written sources [62].

## 4. Conclusions

Dogs undoubtedly played a very important role in the early medieval European society, significantly different from that of other animals. Dog’s meat was consumed in extremely rare cases. Although the use of dog remains was reported (e.g., for the production of bone points or small leather items), it was not common, and it can be assumed that such raw material was used rather reluctantly. Dogs could, however, be used to guard property and herds or to pull sleds. One of their most important functions was to accompany people at home and during hunting. By participating in this type of activities, dogs became an integral part of life for elites and started to be intentionally bred. Certain types were preferred for hunting (dachshund- and greyhound-like) and for home breeding (smaller dogs). This particular function of dogs is also clearly reflected in written sources depicting them in both very positive and negative lights.
